# Mechanistic basis for mitigating drought tolerance by selenium application in tobacco (*Nicotiana tabacum* L.): a multi-omics approach

**DOI:** 10.3389/fpls.2023.1255682

**Published:** 2023-09-19

**Authors:** Huaxin Dai, Jinpeng Yang, Lidong Teng, Zhong Wang, Taibo Liang, Waleed Amjad Khan, Ruiwei Yang, Baoming Qiao, Yanling Zhang, Chunlei Yang

**Affiliations:** ^1^ Department of Tobacco Agriculture, Zhengzhou Tobacco Research Institute of China National Tobacco Corporation (CNTC), Zhengzhou, China; ^2^ Department of Research Center on Tobacco Cultivating and Curing, Tobacco Research Institute of Hubei, Wuhan, China; ^3^ Department of Agronomy, College of Agriculture and Biotechnology, Zhejiang University, Hangzhou, China; ^4^ China Tobacco Gene Research Center, Zhengzhou Tobacco Research Institute of China National Tobacco Corporation (CNTC), Zhengzhou, China; ^5^ Tasmanian Institute of Agriculture, University of Tasmania, Hobart, TAS, Australia

**Keywords:** cigar tobacco, drought, selenium, multi-omics, target genes

## Abstract

The lack of irrigation water in agricultural soils poses a significant constraint on global crop production. In-depth investigation into microRNAs (miRNAs) has been widely used to achieve a comprehensive understanding of plant defense mechanisms. However, there is limited knowledge on the association of miRNAs with drought tolerance in cigar tobacco. In this study, a hydroponic experiment was carried out to identify changes in plant physiological characteristics, miRNA expression and metabolite profile under drought stress, and examine the mitigating effects of selenium (Se) application. The shoot dry weight of drought-stressed plants was approximately half (50.3%) of that in non-stressed (control) conditions. However, plants supplied with Se attained 38.8% greater shoot dry weight as compared to plants with no Se supply under drought stress. Thirteen miRNAs were identified to be associated with drought tolerance. These included 7 known (such as nta-miR156b and nta-miR166a) and 6 novel miRNAs (such as novel-nta-miR156-5p and novel-nta-miR209-5p) with the target genes of *squamosa promoter-binding-like protein 4* (*SPL4*), *serine/threonine protein phosphatase 2A* (*PPP2A*), *cation/calcium exchanger 4-like* (*CCX4*), *extensin-1-like* (*EXT1*) and *reduced wall acetylation 2* (*RWA2*). Further investigation revealed that the expression levels of *Ext1* and *RWA2* were significantly decreased under drought stress but increased with Se addition. Moreover, key metabolites such as catechin and N-acetylneuraminic acid were identified, which may play a role in the regulation of drought tolerance. The integrated analysis of miRNA sequencing and metabolome highlighted the significance of the novel-nta-miR97-5p- *LRR-RLK*- catechin pathway in regulating drought tolerance. Our findings provide valuable insights into the molecular mechanisms underlying drought tolerance and Se-induced stress alleviation in cigar tobacco.

## 1 Introduction

In the past few decades, the Earth’s climate has been seriously impacted by global warming due to increasing levels of greenhouse gases in the Earth’s atmosphere. This has affected normal rainfall patterns, which aggravated drought to extreme levels in many parts of the world, including China. Drought, as a major abiotic constraint on plant growth, has the potential to reduce crop yield by up to 15% (Leng and Hall, 2019). In China, annual losses in agricultural production caused by drought could reach up to 4.2 billion US dollars (USD), equating 0.23% of the national GDP (Su et al., 2018).

Drought stress induces oxidative stress in plants, leading to leaf structure damage and decreased photosynthesis and stomatal activity (Khan et al., 2023). Plants have evolved diverse strategies to cope with the harmful effects of drought, which involve the reprogramming of their transcriptome, proteome and metabolome profiles to enhance stress tolerance. Post-transcriptional regulation by microRNAs (miRNAs) is a crucial regulatory process underlying this reprogramming. The length of miRNAs ranges from 20 to 24 nucleotides in length and these miRNAs are involved in gene silencing in plants (Cuperus et al., 2011), many biological processes, such as plant growth, development, biotic and abiotic stress resistance (Chen, 2005; Cuperus et al., 2011; Teng et al., 2023). Teng et al. (2023) identified 23 differentially expressed miRNAs which were highly associated with Cd tolerance in maize. The target genes of these miRNAs were mainly involved in GSH metabolism, antioxidant enzyme activity and metal transport. Hormones such as abscisic acid and auxin play key roles in regulating drought tolerance in plants. For instance, miR167, which targets auxin response factor, was downregulated by abscisic acid application under drought stress (Liu et al., 2009). Several miRNAs (e.g., miRNA156, miR166, miR167 and miR168) have been demonstrated in certain plant species to regulate the expression of drought-responsive genes, including *SQUAMOSA promoter-binding protein-like* (*SPL*)*, cation/Ca2+ exchanger* (*CCX*)*, REDUCED WALL ACETYLATION* (*RWA*)*, protein phosphatase 2* (*PP2*) *and exostosin glycosyltransferase* (*EXT*) (Gao et al., 2016; Zhang et al., 2018; Feyissa et al., 2019; Liu et al., 2020). Further investigation of drought regulated miRNAs and their target genes would greatly contribute to the understanding of the molecular basis underlying drought-stress tolerance in tobacco.

Tobacco (*Nicotiana tabacum* L.) is an annual or limited perennial herbaceous plant species that belongs to the Solanaceae family. It is a commercial crop extensively cultivated worldwide, including China. In recent years, the global consumption and demands for cigar tobacco have shown steady increase particularly in China and United States (Yan et al., 2021; Wang et al., 2022). Tobacco is primarily cultivated in semi-arid and rain-fed areas, heavily reliant on rainwater to meet the crop water demands. As tobacco is highly susceptible to drought stress, improving tolerance to drought stress in tobacco is of significant economic importance. A comprehensive understanding of drought-responsive transcriptional networks would facilitate the identification of integrated biological pathways involved in mitigating drought stress. Although some key drought responsive miRNAs such as miR160, miR162, miR166, miR168, 390, miR395 and miR398 (Rabara et al., 2015; Yin et al., 2015; Chen et al., 2017) have been identified in tobacco, none of the predicted targets have been further validated through cloning or sequence alignment.

Selenium (Se) is an essential micronutrient in plants and plays a significant role in mitigating the effects of abiotic stress (Cao et al., 2013; Lanza and Dos Reis, 2021). Previous reports have demonstrated the positive effects of Se application on plant drought stress tolerance, including increases in proline and chlorophyll content, accumulation of osmoprotectant and enhancement in antioxidant enzyme activity in plants (Ahmad et al., 2021; Han et al., 2022). Moreover, Se application in plants has been shown to reduce reactive oxygen species (ROS) toxicity under drought stress conditions (Han et al., 2022). Although several studies have reported improved plant performance in tobacco with Se application under drought stress through physiological assessments (Han et al., 2022), the molecular basis underlying this phenomenon remains largely unknown. Therefore, the objectives of this study were: (i) to investigate the negative effect of drought stress on plant growth, (ii) to identify metabolites, miRNAs and prediction of their targeted genes associated with increased drought tolerance, (iii) to demonstrate the effect of Se addition in mitigating plant drought tolerance by inducing the selected miRNAs, target genes and metabolite accumulation in tobacco.

## 2 Materials and methods

### 2.1 Plant materials and growth condition

The commercially grown tobacco cultivar ‘CX26’ is widely used for cigar wrapper production in Hubei Province, China. This cultivar was used in this experiment. Firstly, the seeds were germinated in pots filled with vermiculite and routinely irrigated to maintain adequate moisture levels (Bukhari et al., 2016). Forty-day seedlings were then transplanted to containers containing 12L nutrient solution. The composition of the nutrient solution was set according to Bukhari et al. (2016), which is represented in mg L^-1^: (NH_4_)_2_SO_4,_ 48.2; K_2_SO_4_, 15.9; KNO_3_, 18.5; KH_2_PO_4_, 24.8; MgSO_4_.7H_2_O, 154.88; Ca(NO_3_)_2_.4H_2_O, 86.17; ZnSO_4_.7H_2_O, 0.11; Fe-Citrate, 7.0; CuSO_4_.5H_2_O, 0.04; MnCl_2_.4H_2_O, 0.9; H_2_MoO_4_, 0.01 and H_3_BO_3_, 2.9 (pH, 5.8). Plants were allowed to grow for 7 days in a glasshouse at the Zhengzhou Tobacco Research Institute before the treatments were added. Three treatments (including control) were used in this study: 20% PEG-6000 (Zhang et al., 2017) was added in the nutrient solution for drought stress (DS), with an additional dose of 3 μM Se (as Na_2_SeO_3_, Huang et al., 2021) for Se treatment (DSSe). There was no addition of PEG and Se in control plants (CK). The nutrient solutions were renewed weekly and aerated by using air pumps. Plants were harvested after 6 days of respective treatments and further separated into shoots and roots, by keeping three biological replicates for each treatment. Harvested shoots were immediately stored at -80°C until further use.

### 2.2 Total RNA extraction, library construction and sequencing of small RNA

RNA was extracted from shoot samples using TRIzol reagent (Invitrogen, Carlsbad, CA, USA) according to the manufacturer’s instructions. Nine small RNA libraries were constructed from the collected samples (three samples each per treatment). Total RNA was isolated using small RNA sample pre kit (Illumina, San Diego, California, America) according to the manufacturer’s instructions. The adaptors were added to the small RNAs to reverse transcribe them into cDNA followed by amplification. Further, the PCR products were purified, and the libraries were obtained and quality was assessed using Agilent 2100 Bioanalyzer (Agilent, Palo Alto, CA, USA). Lastly, Illumina sequencing of small RNAs was carried out using HiSeq 4000 (Illumina, San Diego, CA, USA).

### 2.3 Identification of miRNAs

The identification of known and novel miRNAs were performed according to Xu et al. (2013). Briefly, small RNA reads were generated after sequencing and raw data was obtained. The adaptors and low-quality reads were discarded using the ACGT-miR program, and comparisons of clean reads were made using Rfam and GenBank databases. The matched non-coding snRNA, rRNA, tRNA and snoRNA sequences were removed and the remaining clean reads were mapped to the tobacco genome by using Bowtie software v1.1.2 with specific parameter settings according to Langmead et al. (2009). Those sequence reads identical to the known mature miRNAs were selected for subsequent analysis. The unannotated unique miRNAs with fold to stem-loop structure were regarded as novel miRNAs.

### 2.4 Identification of miRNAs in response to drought and selenium treatments with the prediction of target genes

Transcripts per million (TPM) was used to normalize the frequency of miRNAs according to He et al. (2016). Differentially expressed miRNAs (DEMs) between all treatments were detected using DEGseq. The screening criteria were set as: log_2_(DS vs CK) or log_2_(DSSe vs DS) > 1 (upregulated) or <−1 (downregulated), and *p* value ≤0.05. psRNATarget was used to predict target genes of miRNAs (Dai and Zhao, 2011). The prediction of potential target genes for the miRNAs was based on the following criteria: (1) less than four nucleotide mismatches between miRNAs and their target genes; (2) less than three consecutive nucleotide mismatches; and (3) no mismatches at the complementary site between the miRNA sequence and the target mRNA, specifically at positions 10 or 11 of the miRNA sequence, which are considered putative cleavage sites (Kantar et al., 2010).

Total RNA was extracted from stored leaf samples. qRT-PCR was carried out according to Lin et al. (2022). The PCR conditions were comprised of an initial denaturation step at 95°C for 3 min, second denaturation at 94°C for 60 s (40 cycles), annealing at 58°C for 30 s, extension at 72°C for 30 s, and finally extension at 72°C for 5 min. The relative expression level was determined using the ΔΔCT method. The tobacco *NttubA1* was used as a reference gene. The primers of *NtRWA2* and *NtEXT1* were listed in **Table S1**.

### 2.5 Metabolomic analysis

The extraction, identification and quantification of leaf metabolites were carried out according to Lin et al. (2022). Briefly, the samples were placed in tubes containing 1 ml extraction solution (methanol: acetonitrile: water, 2: 2: 1). After 40 Hz trituration for 4 min and 5 min sonification, the sample extracts were centrifuged at 12,000 rpm at 4°C for 20 min and supernatants were collected and further analyzed using a liquid chromatography–mass spectrometry (LC-MS) system. The metabolites were separated by a UHPLC system with a UPLC HSS T3 column. Briefly, the chromatographic separation was carried out using Hypersil GOLD aQ Dim column (Thermo Fisher Scientific, USA). The mobile phase consisted of a 0.1% formic acid aqueous solution (A solvent) and acetonitrile containing 0.1% formic acid (B solvent). The elution was conducted using the following gradient: 0-2 minutes, 5% B solvent; 2-22 minutes, 5-95% B solvent; 22-27 minutes, 95% B solvent; 27-27.1 minutes, 95-5% B solvent; 27.1-30 minutes, 5% B solvent. The flow rate was set at 0.3 ml min^-1^. The temperature of column was set at 40°C, and the volume of injection was 5 μl. The metabolites were detected using a high-resolution tandem mass spectrometer. The data of mass spectrometry was then imported into Compound Discoverer 3.2 (Thermo Fisher Scientific, USA) software. By mapping the BMDB (BGI Metabolome Database, China), mzCloud database, and ChemSpider database, a data matrix that included information such as metabolite peak areas and identification results was obtained. Metabolites with fold change > 1.20 or < 0.83, *P* value < 0.05, were regarded as differentially accumulated metabolites, which were monitored in DS vs CK and DSSe vs DS treatments.

### 2.6 Statistical analysis

The illustration of graphs was performed using Origin software (Origin Lab Corporation, Wellesley Hills, Wellesley, MA, USA) and statistical analyses were conducted using Data Processing System (DPS) statistical software. One-way ANOVA multiple comparisons and Duncan’s multiple range test were used to compare the means of all the treatments used in this study. The significance of all tests was determined at a level of *P* < 0.05.

## 3 Results

### 3.1 Effects of drought stress and selenium treatment on plant growth

**Figure 1:**
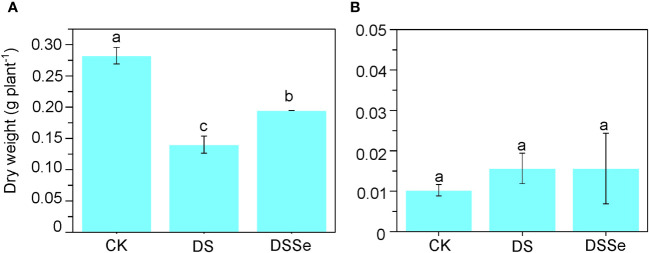
Exogenous application of Se drought stress tolerance in tobacco. The dry weight of shoot **(A)** and root **(B)** of CX26 grown under control (CK), drought stress (DS) and drought stress+Se (DSSe) conditions after 40 DAS. Drought stress was applied by adding 20% PEG-6000 into plant nutrient solution, while additional dose of 3 μM Na2SeO3 was supplied to drought + Se treatment. Data are presented as mean ± SD (n = 3). Different letters indicate statistically significant differences. DAS, days after sowing; Se, selenium; PEG, polyethylene glycol.

Drought stress markedly inhibited plant growth after 6 days of stress application (**Figure 1**) with the seedlings exhibiting clear symptoms of leaf chlorosis. The shoot dry weight of DS seedlings was only 50.3% of that of non-stressed (CK) seedlings (**Figure 1**), albeit no significant difference was found in their root dry weight. The shoot dry weight of DSSe seedlings was 38.8% higher than that of DS seedlings (**Figure 1**), with no significant differences were recorded in root dry weight.

### 3.2 Analysis of small RNAs

The differences in plant growth under different treatments led to the identification of drought tolerance-related miRNAs and their target genes. A total of 36,045,627, 36,604,788 and 35,979,733 raw reads were generated from the CK, DS and DSSe treatments, respectively (**Table 1**) and 35,124,114 (97.44%), 35,779,457 (97.75%) and 35,278,102 (98.05%) reads were remained after filtering and trimming of the CK, DS and DSSe groups, respectively. Of the high-quality sequence reads obtained, 95.38%, 95.36% and 95.37% of the CK, DS and DSSe groups were mapped to the tobacco genome (**Table 1**). In total, 1,006,917 (2.79%), 994,429 (2.72%) and 1,048,264 (2.91%) miRNA reads were mapped to the reference genome in the CK, DS and DSSe treatments, respectively. Of these, 142, 138 and 135 were conserved miRNAs, whereas 319, 302 and 309 were novel miRNAs identified in control, DS and DSSe treatments, respectively (**Table 1**).

**Table 1:** Summary of high-throughput sequencing of small RNAs from tobacco shoots.

	CK^1^	DS	DSSe
Total raw reads	36,045,627	36,604,788	35,979,733
Total clean reads	35,124,114	35,779,457	35,278,102
	(97.44%)	(97.75%)	(98.05%)
Mapped reads	33,500,000	34,120,000	33,643,333
	(95.38%)	(95.36%)	(95.37%)
Total miRNA reads	1,006,917	994,429	1,048,264
	(2.79%)	(2.72%)	(2.91%)
Conserved miRNAs	142	138	135
Novel miRNAs	319	302	309

^1^ CK, DS and DSSe correspond to tobacco grown in basic nutrient solution (BNS), BNS with 20% PEG, BNS with 20% PEG + 3 μM Na_2_SeO_3_, respectively.

**Figure 2:**
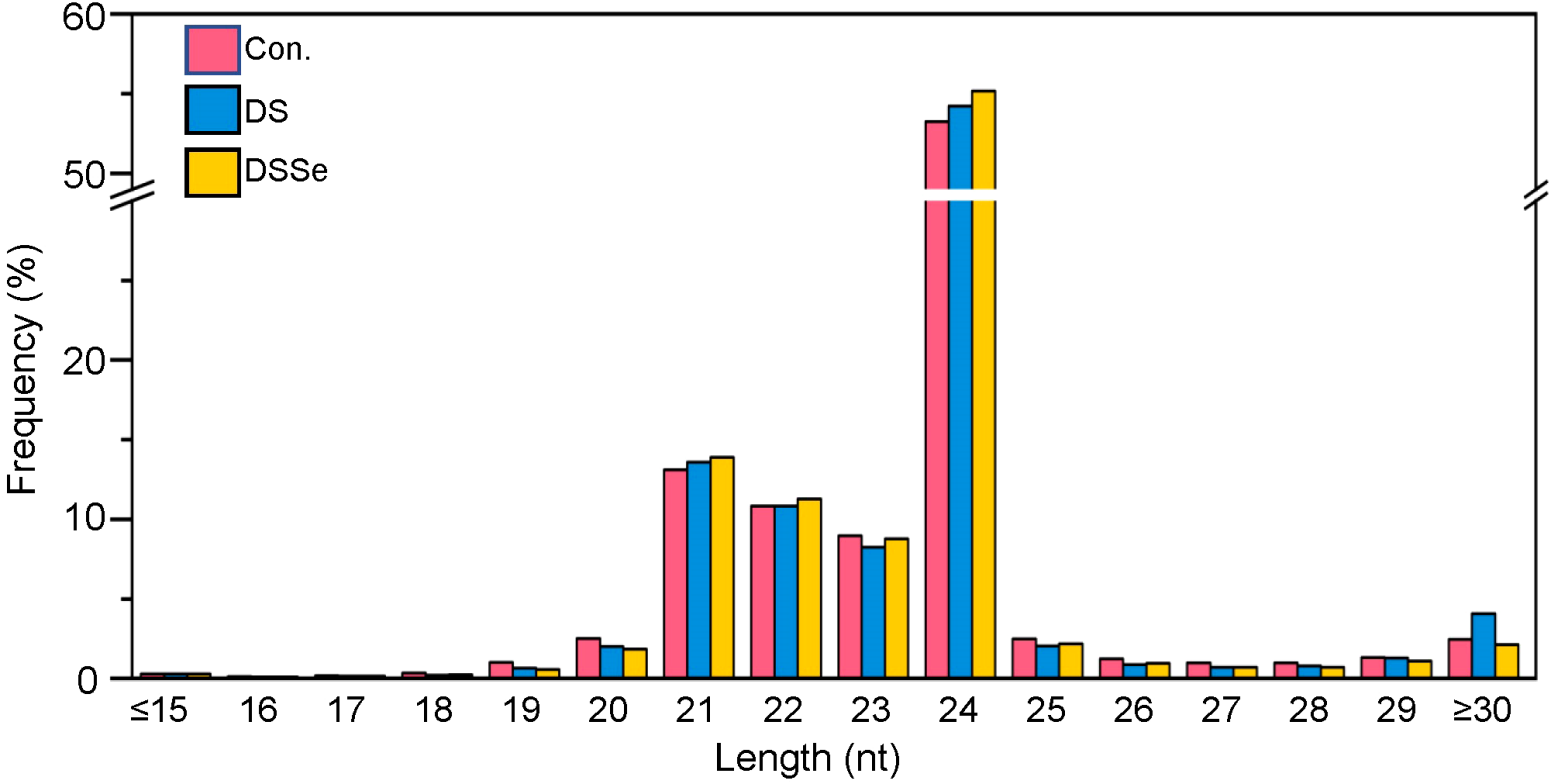
Length distribution of small RNAs in shoots of CX26. The X-axis represents the small RNA length (nucleotide) and the Y-axis represents the percentage of small RNA reads. Control (pink), DS (blue) and DSSe (yellow) represent control, drought (20% PEG-6000 treatment) and drought + 3 μM Na_2_SeO_3_.

**Figure 3:**
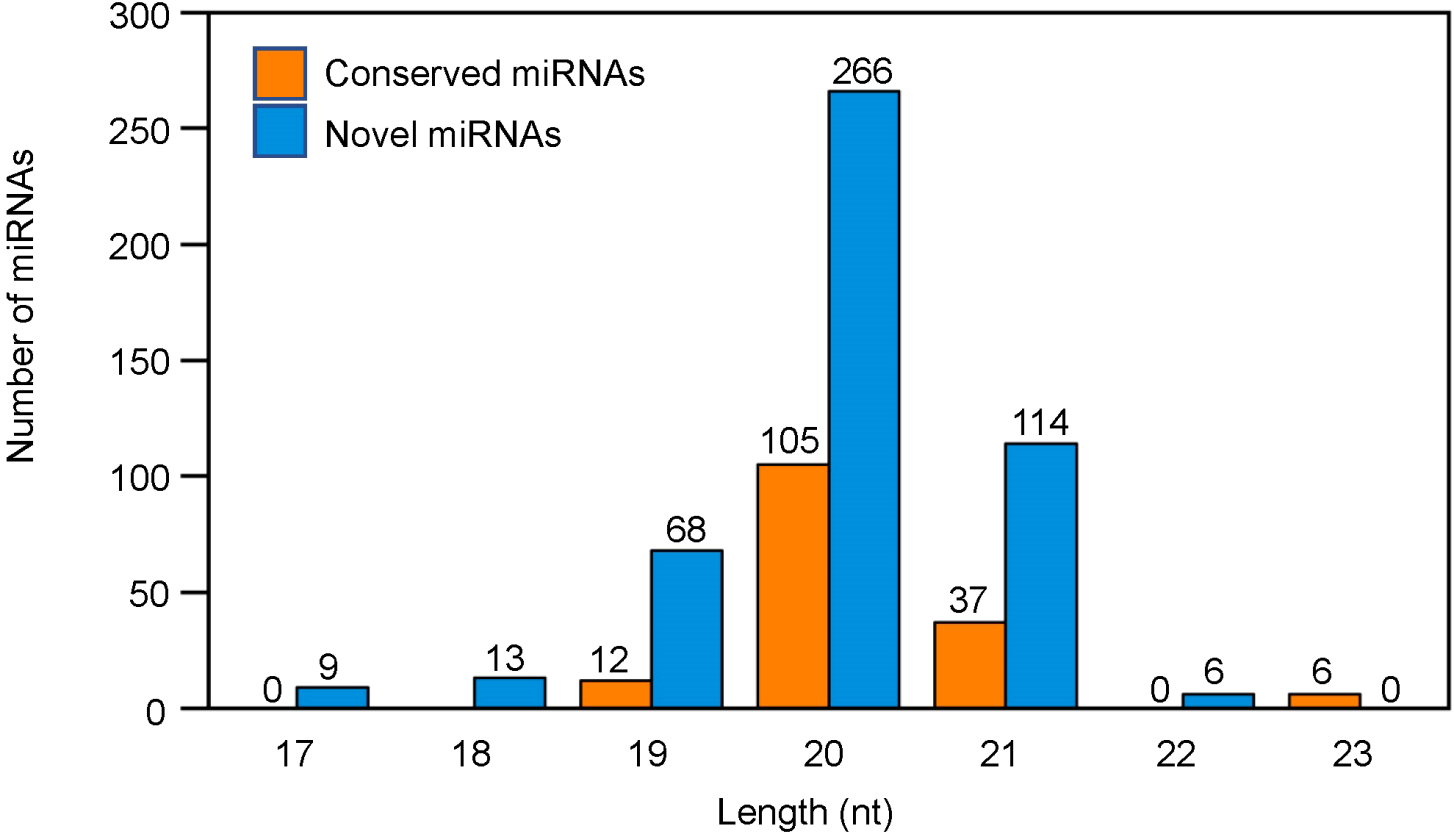
Length based distribution of conserved and novel microRNAs (miRNAs) in tobacco shoots.

Regarding the length distribution of miRNAs, the 21-24 nt sequences were found to be dominant with 24 nt sequences being the most abundant length amongst all the treatments (**Figure 2**). The conserved miRNAs were mainly distributed at 20 nt and 21 nt, whereas the novel miRNAs were mainly distributed at 19 nt, 20 nt and 21 nt (**Figure 3**).

### 3.3 Analysis of differentially expressed miRNAs under different treatments

**Figure 4:**
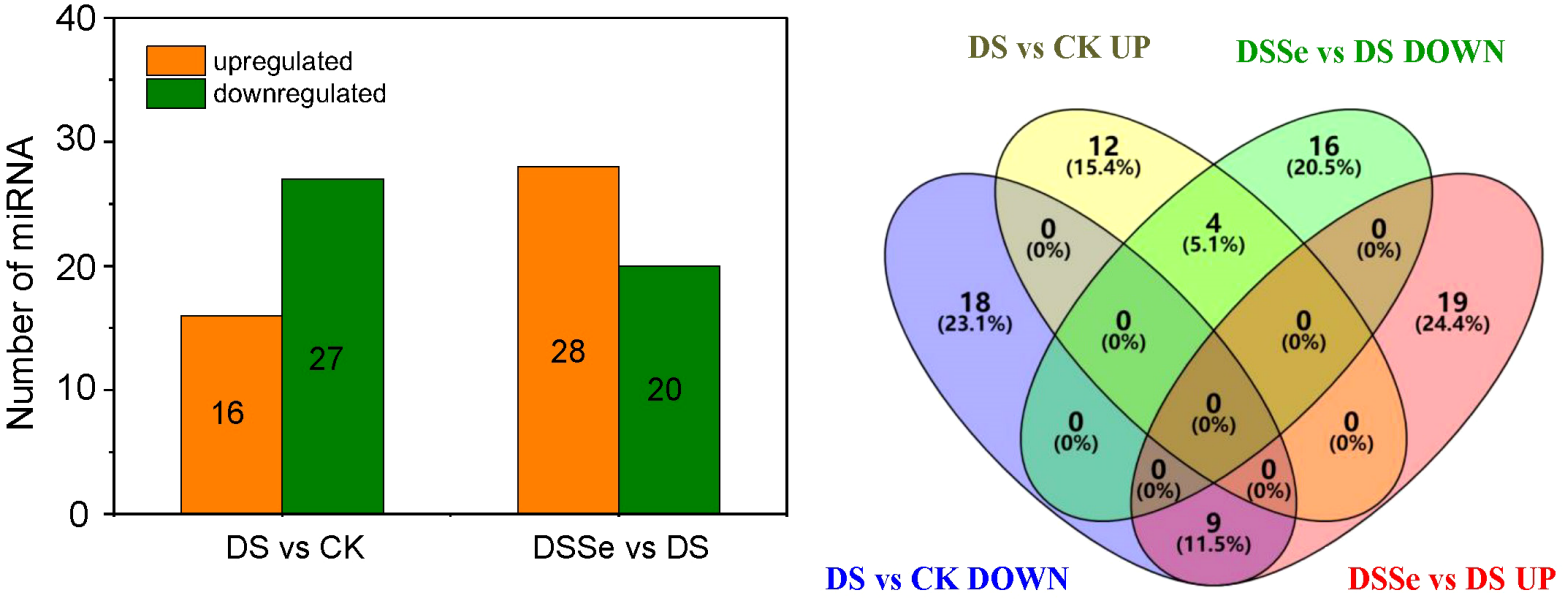
Differential expression miRNAs under DS and DSSe treatments. The CK, DS and DSSe represent control, drought and drought+Se.

The miRNA expression profiles were greatly influenced by the growth condition with 16 and 27 miRNAs being upregulated and downregulated, respectively, under drought treatment as compared to the control (**Figure 4**, **Tables S1**, **S2**). Among the downregulated miRNAs, 12 were known and 15 were novel. These novel miRNAs included miRNA156, miR166, miR167 and miR168 family miRNAs (**Table S1**). More than 75% (10) of the upregulated miRNAs were known, with only 3 novel miRNAs (**Table S2**). Those known miRNAs belonged to 12 miRNA families. Moreover, 28 upregulated and 20 downregulated miRNAs were detected in the DSSe as compared to DS (**Tables S3**, **S4**). Amongst 20 downregulated miRNAs identified, 11 were known and 9 were novel (**Table S3**). The downregulated miRNAs between DSSe and DS consisted of nta-miR156a (25.0-fold), nta-miR399f (24.7-fold) and novel-nta-miR27-5p (22.8-fold). Further, we recorded 12 known and 16 novel upregulated miRNAs in the DSSe as compared to DS and these included miR156b, miR156d, miR166a and miR390a (**Table S4**).

**Figure 5:**
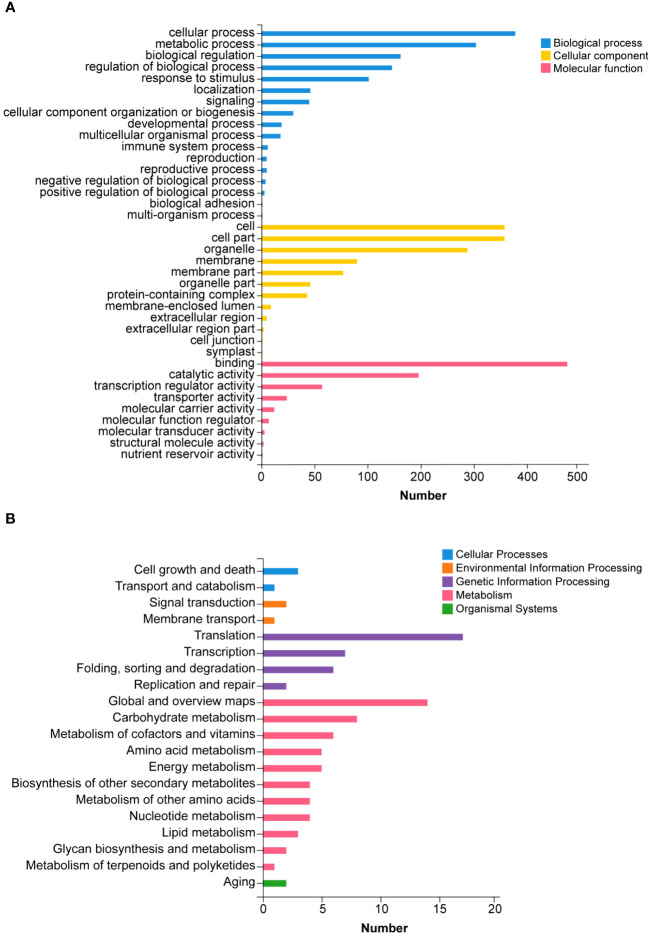
Gene Ontology (GO) **(A)** and Kyoto Encyclopedia of Genes and Genomes (KEGG) **(B)** analysis for target genes of differently expressed miRNA under different conditions in tobacco.

Interestingly, nine miRNAs were downregulated in DS vs CK but upregulated in DSSe vs DS (Group I, **Figures 4**, **5**). These miRNAs were comprised of six novel (novel-nta-miR156-5p, novel-nta-miR209-5p, novel-nta-miR248-5p, novel-nta-miR345-3p, novel-nta-miR378-3p and novel-nta-miR97-5p) and three known miRNAs (nta-miR156b, nta-miR166a and nta-miR482b-3p). Furthermore, nta-miR156b was found to be downregulated (-24.5-fold) in DS but upregulated (22.8-fold) in DSSe (**Table 2**). Meanwhile, four miRNAs were upregulated in DS vs CK, while the four miRNAs were downregulated in DSSe vs DS (Group II, **Table 3**). All those miRNAs were known, including nta-miR156c, nta-miR482c, nta-miR6019b and nta-miR6154a. The miRNAs of Group I and II may play key roles in Se-induced drought alleviation.

**Table 2:** List of miRNAs downregulated under DS treatment (DS vs control) but upregulated under DSSe treatment (DSSe vs D).

Gene ID	log_2_ (DS/CK)	Q value	log_2_ (DSSe/DS)	Q value	Target Gene ID	Annotation
novel-nta-miR156-5p	-2.2	0.001	1.57	3.46E-03	LOC107771768	transcription factor bHLH30-like
					LOC107796775	metal-nicotianamine transporter YSL7
novel-nta-miR209-5p	-1.8	0.043	1.91	1.34E-03	LOC107808871	serine/threonine-protein kinase RUNKEL
					LOC107808555	D-3-phosphoglycerate dehydrogenase 2,
novel-nta-miR248-5p	-7.1	0.003	5.31	7.38E-03	LOC107766225	protein DETOXIFICATION 49-like
novel-nta-miR345-3p	-8.9	0.016	8.86	1.02E-02	LOC107764608	SPX domain-containing membrane protein At4g22990-like
novel-nta-miR378-3p	-1.7	0.036	2.12	4.09E-07	LOC107790606	cation/calcium exchanger 4-like
novel-nta-miR97-5p	-2.7	0.000	1.49	1.69E-02	LOC107761676	LRR receptor-like serine/threonine-protein kinase
nta-miR156b	-24.5	0.000	22.82	3.20E-07	LOC107762327	squamosa promoter-binding-like protein 4
nta-miR166a	-9.2	0.004	9.44	3.32E-03	LOC107760551	homeobox-leucine zipper protein ATHB-15-like
nta-miR482b-3p	-8.8	0.003	9.65	4.71E-05	LOC107760541	putative late blight resistance protein homolog R1A-10

**Table 3:** List of miRNAs upregulated under drought treatment (DS vs CK) but downregulated under DSSe treatment (DSSe vs D).

Gene ID	log_2_ (DS/CK)	Q value	log_2_ (DSSe/DS)	Q value	Target Gene ID	Annotation
nta-miR156c	7.66	0.00	-5.74	2E-02	LOC107770163	serine/threonine protein phosphatase 2A 55 kDa regulatory subunit B beta isoform-like
nta-miR482c	7.89	0.01	-9.78	2E-04	LOC107775023	TMV resistance protein N-like
nta-miR6019b	10.42	0.01	-11.35	4E-02	LOC107778652	extensin-1-like
nta-miR6154a	13.56	0.00	-13.54	3E-04	LOC107800019	protein REDUCED WALL ACETYLATION 2

### 3.4 Identification of target genes and function categories of DEMs

**Figure 6:**
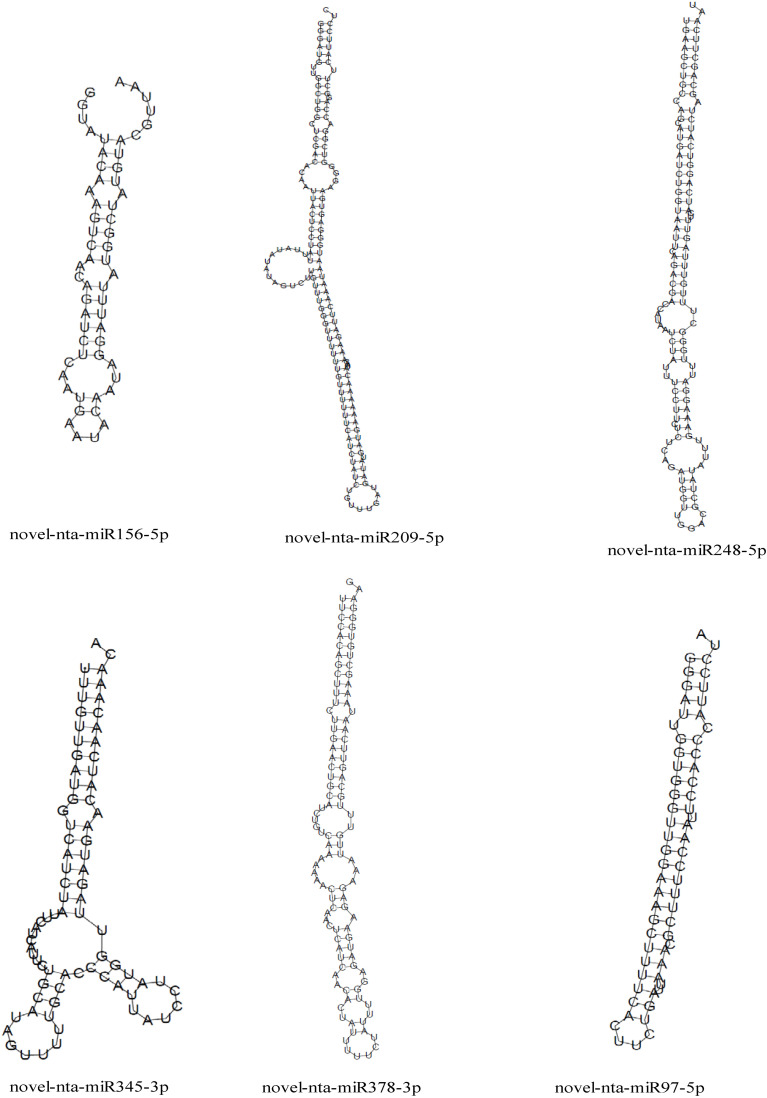
Precursor secondary structure prediction of 6 novel miRNAs.

We predicted the target genes of the identified miRNAs using psRNATarget. Gene Ontology (GO) analysis showed that these target genes are mainly involved in cellular process, metabolic process, cell, cell part, organelle, binding and catalytic activity (**Figure 5A**). Kyoto Encyclopedia of Genes and Genomes (KEGG) analysis revealed that these target genes were involved in translation, transcription and carbohydrate metabolism (**Figure 5B**). Moreover, these target genes were involved in cellular processes, environmental information processes and organismal systems (**Figure 5B**). We classified the resultant target genes into Groups I and II based on their predicted functions. Group I contained eleven target genes, including transcription factors, metal transporters, protein kinases and detoxification-related proteins (**Table 2**), with the identification of three transcription-related genes e.g., *transcription factor bHLH30-like, squamosa promoter-binding-like protein 4* (S*PL4*) and *homeobox-leucine zipper protein ATHB-15-like*. We also detected two metal transporters (*metal-nicotianamine transporter YSL7* and *cation/calcium exchanger 4-like SlCCX4*) and two protein kinase genes (*serine/threonine-protein kinase RUNKEL* and *LRR receptor-like serine/threonine-protein kinase*). The other four genes included *D-3-phosphoglycerate dehydrogenase 2, protein DETOXIFICATION 49-like, SPX domain-containing membrane protein At4g22990-like* (*SPX*) and *late blight resistance protein homolog R1A-10*. In Group II, the targets of the four miRNAs were *serine/threonine protein phosphatase 2A 55 kDa regulatory subunit B beta isoform-like* (*PP2A*)*, TMV resistance protein N-like, extensin-1-like* (*EXT1*) and *Reduced Wall Acetylation 2* (*RWA2*). The putative secondary structures of the six novel miRNAs were also predicted (**Figure 6**).

**Figure 7:**
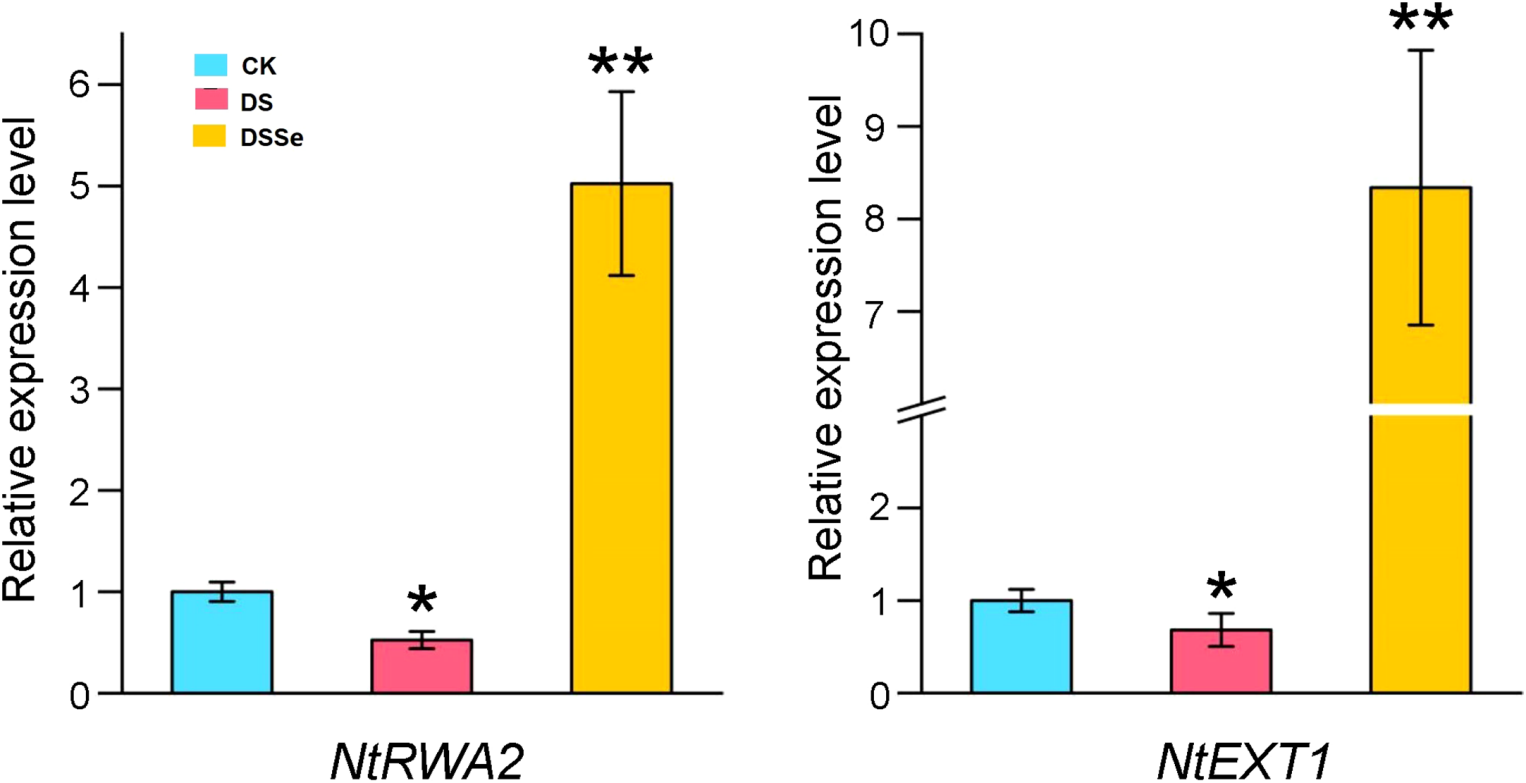
Relative expression of *NtRWA2* and *NtEXT1* under DS and DSSe treatments. * and ** represent significant difference at P <0.05 and 0.01, respectively.

To further investigate whether the differentially expressed miRNA target genes were involved in drought tolerance, we selected two target genes (*NtRWA2* and *NtEXT1*) for qRT-PCR analysis. The relative gene expression of *NtRWA2* and *NtEXT1* was significantly decreased in DS vs CK and inceased in DSSe vs DS (**Figure 7**). These results suggested that *NtRWA2* and *NtEXT1* might play an important function in regulating drought tolerance.

### 3.5 Analysis of differentially accumulated metabolites under different treatments

This study recorded 130 upregulated and 77 downregulated DAMs after drought treatment (DS vs CK), while there were 64 upregulated and 63 downregulated DAMs after the addition of Se (DSSe vs DS) (**Figure S1**). Interestingly, eight metabolites including ur-144 n-(2-hydroxypentyl) metabolite and o-[(2e)-hexenedioyl] carnitine were downregulated in DS treatment but were upregulated in DSSe treatment (**Table 4**). We also identified 14 metabolites were upregulated in DS treatment but downregulated in DSSe treatment, which included 9,10-dibromostearic acid, quinoline-3-carboxamides, lactose, N-acetylneuraminic acid and catechin (**Table 5**). These DAMs might play key roles in drought tolerance in tobacco.

**Table 4:** List of metabolites downregulated under drought treatment (DS vs CK) but upregulated under DSSe treatment (DSSe vs D).

Metabolites	log_2_ (DS/CK)	*P* Value	log_2_ (DSSe/DS)	*P* Value
Ur-144 n-(2-hydroxypentyl)	0.49	0.02	1.48	0.03
O-[(2e)-hexenedioyl]carnitine	0.49	0.05	1.78	0.02
N-2-ethylhexyl bicycloheptenedicarboximide	0.33	0.01	3.20	0.01
N-(tetrahydrofuran-2-ylmethyl)quinoline-8-sulfonamide	0.29	0.01	2.31	0.03
1,4:3,6-dianhydro-2-deoxy-5-o-[(4-isopropylphenyl)carbamoyl]-2-[(4-methylbenzoyl)amino]-d-glucitol	0.28	0.02	2.28	0.05
2-[(3s)-1-benzyl-3-pyrrolidinyl]-1-methyl-1h-benzimidazole	0.24	0.04	1.50	0.04
N,N-diethyl-9-oxobicyclononane-3-carboxamide	0.14	0.02	5.86	0.01
phenyl hydrogen sulfate	0.04	0.05	3.15	0.03

phenyl hydrogen sulfate, 2-hydroxy-5-[3-(4-methoxy-1-benzofuran-5-yl)-3-oxopropanoyl]phenyl hydrogen sulfate.

**Table 5:** List of metabolites upregulated under drought treatment (DS vs CK) but downregulated under DSSe treatment (DSSe vs D).

Metabolites	log_2_ (DS/CK)	P Value	log_2_ (DSSe/DS)	P Value
9,10-dibromostearic acid	21.64	0.00	0.04	0.00
Quinoline-3-carboxamides	17.39	0.04	0.05	0.02
Lactose	14.34	0.02	0.14	0.05
Oglufanide	6.71	0.01	0.27	0.01
Catechin	3.09	0.02	0.39	0.02
Butabarbital	3.05	0.03	0.26	0.02
Rhusflavanone	2.75	0.01	0.42	0.01
N-acetylneuraminic acid	2.42	0.05	0.19	0.00
Mfcd12912432	2.33	0.01	0.47	0.03
Dl-arginine	1.84	0.02	0.40	0.01
Homosalate	1.49	0.01	0.69	0.02
13,14-dihydro-15-keto-tetranor prostaglandin d2	1.49	0.03	0.70	0.03
(25s)-11alpha,20,26-trihydroxyecdysone	1.37	0.04	0.72	0.03
T-2 triol	1.37	0.04	0.62	0.03

## 4 Discussion

**Figure 8:**
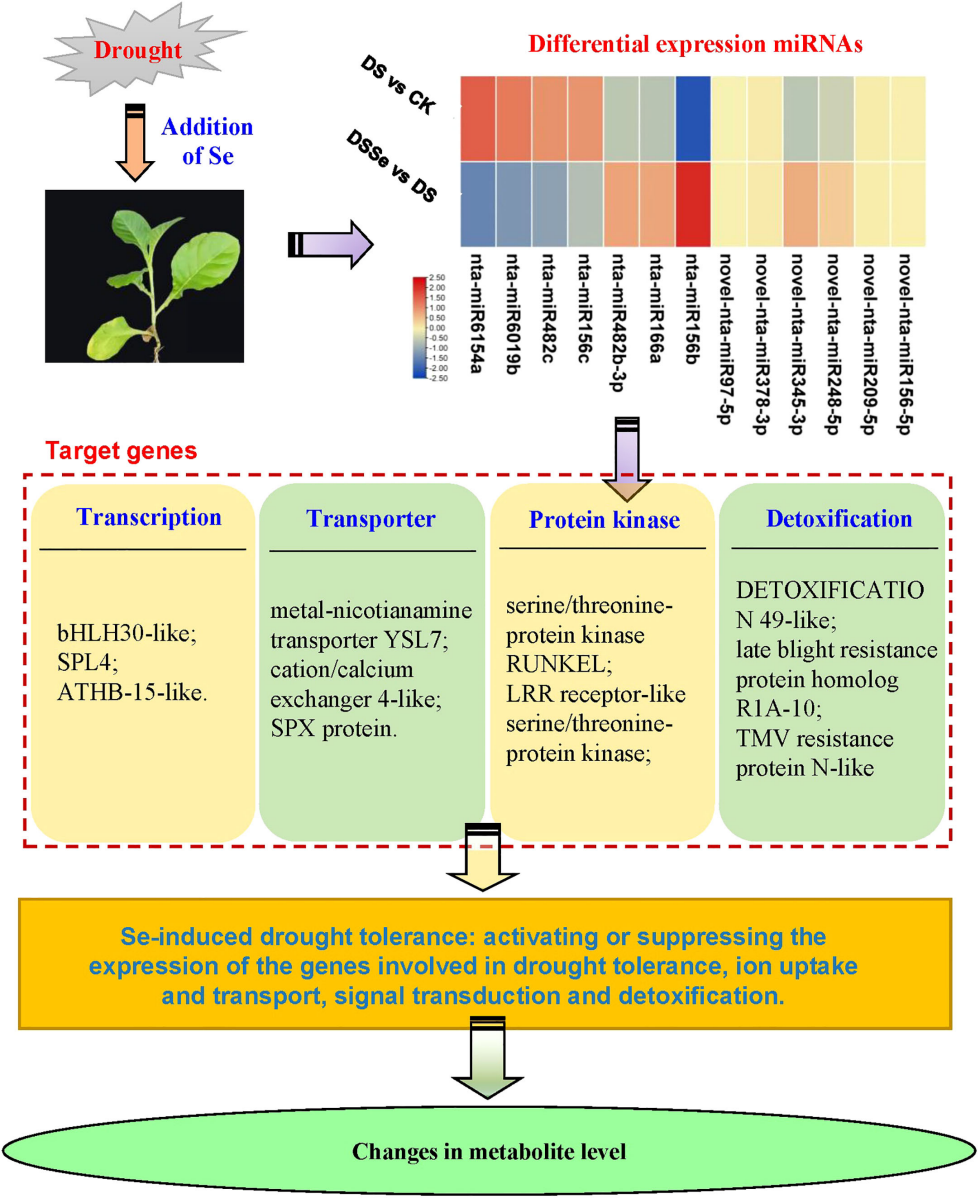
A working model of miRNAs and their target genes in tobacco involving in Se-induced increased drought tolerance. ATHB-15-like, homeobox-leucine zipper protein ATHB-15-like; bHLH30-like, transcription factor bHLH30-like; SPX protein, SPX domain-containing membrane protein; SPL4, squamosa promoter-binding-like protein 4.

This study revealed differences between non-stressed and drought-stressed plants in their biomass production, miRNA expression and metabolite profiling. In addition, these differences were better visualized and understood by adding another treatment of Se, which helped to reduce the yield losses under drought conditions. Our results demonstrated that Se application can be used as a useful strategy to improve drought tolerance in tobacco. Recently, Han et al. (2022) compared the alleviation effects of selenite on drought stress in tobacco and found that different Se species could alleviate drought-induced growth inhibition via elevating photosynthesis, osmotic substance content, antioxidant enzyme activity and stress-responsive genes. However, the regulatory mechanisms of miRNAs conferring drought tolerance in tobacco are largely unknown. Our study has highlighted several important miRNAs that may be involved in regulating drought stress tolerance in tobacco. Based on the identified miRNAs and DAMs, we proposed a tentative model involving drought tolerance (**Figure 8**).

Establishing a correlation between miRNAs and metabolites could provide a better understanding of Se-induced drought tolerance in tobacco and identify key genes for drought tolerance. In our study, we identified 2 miRNAs that showed significant positive or negative correlations with 6 metabolites (**Table S6**, **Tables 2**-**5**). The target genes of these miRNAs were *metal-nicotianamine transporter YSL7* (*YSL7*) and *LRR receptor-like serine/threonine-protein kinase* (*LRR-RLK*). Previous studies have reported the involvement of *OsYSL2* in the phloem transport of Fe and Mn in rice (Koike et al., 2004) and *ZmYSL2* for iron distribution and development in maize (Zang et al., 2020). Additionally, *OsYSL9* has been found to play a role in the distribution of Fe in developing rice grains (Senoura et al., 2017). In the present study, the novel-nta-miR156-5p exhibited significantly positive and negative correlation with phenyl hydrogen sulfate and lactose, respectively (**Table S6**). Similiarly, the novel-nta-miR97-5p exhibited negative correlations with butabarbital, catechin and oglufanide, and its target gene, *LRR-RLK*, has been implicated in regulating abiotic stress tolerance through ABA signaling activation and ROS detoxification (Ye et al., 2017). Catechin, a flavonoid, has been previously reported to enhance flooding tolerance by increasing the capacity of ROS scavenging (Yiu et al., 2011). Henschel et al. (2023) found that foliar application of L-carnitine significantly alleviated drought-induced growth inhibition by elevating membrane integrity and water balance in radish plants. In the present study, the abundance of carnitine was notably decreased under drought stress, but increased after the addition of Se, suggesting a potential positive correlation between carnitine and drought tolerance in tobacco. The accumulation of tea catechins have been found to be improved under drought stress (Lv et al., 2021). The possible mechanism by which catechin responds to drought stress may involve clearing over-accumulated reactive oxidative species (Lv et al., 2021), which is in agreement to the findings of our study. However, the abundance of catechins was found to be decreased after Se addition. This might be due to lower accumulation of ROS in plants under DSSE as compared to DS conditions. These results suggested that the novel-nta-miR97-5p- *LRR-RLK*- catechin pathway might play a central role in drought tolerance, which needs to be further validated in future studies.

Extensin and reduced wall acetylation proteins play numerous functions in plant cell wall (CW) (Manabe et al., 2013). Extensin is the most abundant protein in plant CW and has an important function in plant defense via strengthening the CW (Castilleux et al., 2021). The expression of *extensin-like gene* is significantly induced by drought stress in tolerant wheat genotypes but downregulated in sensitive genotypes under similar circumstances (Keskin, 2019). Manabe et al. (2013) found that the growth of the triple and quadruple *rwa* mutants was severely inhibited through affecting the cell differentiation of secondary CW, thus showing the significance of reduced wall acetylation (RWA) proteins in CW acetylation of plants. In the present study, the expression of *NtRWA2* and *NtEXT1* was significantly decreased in DS treatment but significantly increased in DSSe treatment (**Figure 7**), which reflects their importance in regulating drought tolerance in tobacco. The results presented in this study needs to be tested by conducting further studies for the better understanding of the drought tolerance mechanism in tobacco.

Several transcription factors regulate gene expression during plant growth and development when grown under stress conditions (Lin et al., 2022). Overexpression of *bHLH55* increases salt tolerance in maize (Yu et al., 2021). On the contrary, the knockout of *OsbHLH24* enhances salt tolerance in rice. Compared with wild type plants, the mutant plants have higher scavenging capacity of reactive oxygen species. Moreover, knock-out of *OsbHLH24* also markedly upregulated salt tolerance-related genes, such as *OsSOS1*, *OsHAK7* and *OsHKT1;3* (Alam et al., 2022). In addition, the *SPL* gene family is involved in the activation of the conserved miR156-*SPL* module, which is a key regulator of plant abiotic stress resistance (Wang et al., 2019). Overexpression of *MtHB2* (a homeobox-leucine zipper protein) in transgenic *Arabidopsis* leads to a greater more sensitivity to salt and drought compared with the wild type (Song et al., 2012). In this study, the expressions of novel-nta-miR156-5p, nta-miR156b and nta-miR166a was significantly downregulated in DS treatment but upregulated in DSSe treatment (**Table 2**). Their targets were *transcription factor bHLH30-like, squamosa promoter-binding-like protein 4* and *homeobox-leucine zipper protein ATHB-15-like*. These results suggested that the transcription factors may negatively regulate drought tolerance and Se addition can inhibit the expression of these genes via regulating miRNA expression.

Drought stress affects the uptake and transport of nutrient element (Lanza and Dos Reis, 2021). The cation/calcium exchanger (CCX) family is involved in the transport of Ca and other cations (Su et al., 2021). Yang et al. (2021) found that apple *MdCCX2* positively regulated salt tolerance by decreasing Na^+^ content and elevating the ability of ROS scavenge. SPX proteins play a negative role in regulating phosphorus signaling and are involved in inhibiting the phosphorus starvation response by *PHR* genes (Zhang et al., 2021). In this study, several members of *metal-nicotianamine transporter* (*YSL*)*, CCX* and *SPX* family target genes were identified in tobacco (**Table 2**). Our results suggested that the target genes may elevate drought tolerance via regulating the uptake, transport and distribution of nutrient elements, such as phosphorus. Further research needs to be carried out in future to investigate how miRNA and target genes regulate these processes.

This study identified two protein kinase genes as miRNA targets, *serine/threonine-protein kinase RUNKEL* and *LRR-RLK* (**Table 2**). Their corresponding miRNAs were novel-nta-miR209-5p and novel-nta-miR97-5p. Previous studies found that serine/threonine-protein kinases were involved in abiotic stress tolerance (Luo et al., 2006). The above differentially expressed miRNAs may negatively regulate drought tolerance by inhibiting the two protein kinases. Meanwhile, we also found four miRNAs that were upregulated in DS and downregulated in DSSe treatment (**Table 3**). One of their targets was *serine/threonine protein phosphatase 2A (PP2A) 55 kDa regulatory subunit B beta isoform-like*. *PP2A* also positively regulates abiotic stress tolerance in wheat (Liu et al., 2019). These miRNAs and their target modules may positively regulate drought tolerance in tobacco.

This study revealed 22 differentially accumulated metabolites, which might be associated to regulate drought tolerance. We suggest that further research needs to be carried out to investigate the relationship between the identified DAMs and drought tolerance, and how miRNA operates for the activation of metabolite pathways that led to their increased accumulation.

## 5 Conclusions

Selenium application significantly alleviated drought-induced growth inhibition. The present study identified seven known and six novel miRNAs in tobacco seedlings associated with Se-induced drought stress alleviation, including nta-miR156c, nta-miR482c, nta-miR6019b and nta-miR6154a. Functional analysis of the identified target genes revealed that these miRNAs belonged to transcription factors, metal transporters, protein kinase, etc. Twenty-two key metabolites were identified to be involved in drought tolerance. The combined analysis of miRNA sequencing and metabolome identified a metabolic pathway associated with regulating drought tolerance. Thus far, this is the first study to investigate the effect of exogenous Se application in regulating plant drought-tolerance in tobacco using a multi-omics strategy. The implications of this research helped to better understand the physiological and molecular basis underlying Se mediated defense mechanisms in tobacco. The identified miRNAs and their targets can be used in future to achieve greater drought tolerance in tobacco.

## Data availability statement

The original contributions presented in the study are included in the article/**Supplementary Material**. Further inquiries can be directed to the corresponding authors.

## Author contributions

HD: Conceptualization, Writing – original draft, Writing – review & editing, Data curation. JY: Conceptualization, Methodology, Writing – original draft. LT: Visualization, Writing – original draft. ZW: Data curation, Writing – original draft. TL: Data curation, Formal Analysis, Writing – original draft. WK: Data curation, Writing – review & editing. RY: Formal Analysis, Investigation, Writing – original draft. BQ: Investigation, Writing – original draft. YZ: Resources, Supervision, Writing – review & editing. CY: Project administration, Resources, Writing – review & editing.
