# Supplementary material for: Mechanistic basis for mitigating drought tolerance by selenium application in tobacco (Nicotiana tabacum L.): a multi-omics approach
Source: Front Plant Sci. 2023 Sep 19;14:1255682. doi: 10.3389/fpls.2023.1255682 (PMC10548829; doi:10.3389/fpls.2023.1255682)
Supplement: Supplementary file 1 [file DataSheet_1.docx]

Supplementary Material

# Supplementary Figures and Tables

## Supplementary Figures


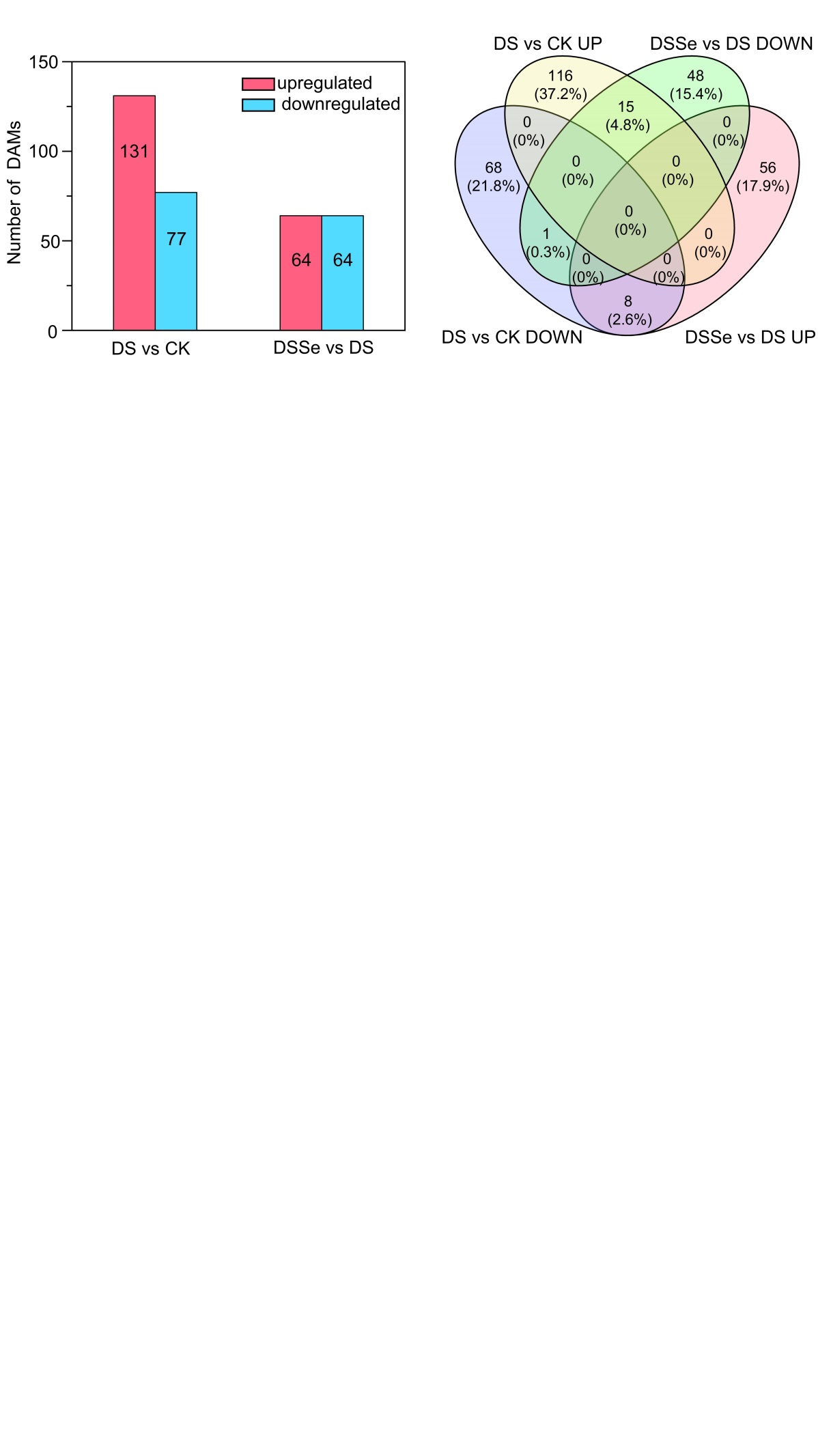


**Supplementary Figure 1.** Differential accumulated metabolites under DS and DSSe treatments. The CK, DS and DSSe represent control, drought and drought + Se.

## Supplementary Tables

**Table S1.** Primers were used in this study

| **Primer name** | | **Primer Sequence（5’- 3’）** |
| --- | --- | --- |
| *NtEXT1-*qPCR | Forward | AAAGCTCAGGAAAGCTGGGG |
|  | Reverse | TGCGAGGGTTGGAAATTTGT |
| *NtRWA2-*qPCR | Forward | TGAAGTGGTTTGGAGCCCAT |
|  | Reverse | AAGACCTGAACGGAACTGCC |
| *NtTubA1*-qPCR | Forward | CAAGACTAAGCGTACCATCCA |
|  | Reverse | TTGAATCCAGTAGGGCACCAG |

**Table S2.** List of miRNAs down-regulated under drought treatment (D VS control)

| **Gene ID** | **log_2_ (D / CK)** | **Q value** | **Target Gene ID** | **Annotation** |
| --- | --- | --- | --- | --- |
| novel-nta-miR107-3p | -6.8 | 0.016 | LOC107831644 | disease resistance protein TAO1-like |
| novel-nta-miR156-5p | -2.2 | 0.001 | LOC107796775 | probable metal-nicotianamine transporter YSL7 |
| novel-nta-miR209-5p | -1.8 | 0.043 | LOC107808555 | D-3-phosphoglycerate dehydrogenase 2 |
| novel-nta-miR248-5p | -7.1 | 0.003 | LOC107766225 | protein DETOXIFICATION 49-like |
| novel-nta-miR343-3p | -5.2 | 0.002 | LOC107814379 | gibberellin 2-beta-dioxygenase 8-like |
| novel-nta-miR345-3p | -8.9 | 0.016 | LOC107764608 | SPX domain-containing membrane protein At4g22990-like |
| novel-nta-miR378-3p | -1.7 | 0.036 | LOC107790606 | cation/calcium exchanger 4-like |
| novel-nta-miR38-5p | -1.8 | 0.036 | LOC107809110 | endochitinase A-like |
| novel-nta-miR40-3p | -1.5 | 0.036 | LOC107797283 | basic transcription factor 3-like |
| novel-nta-miR429-5p | -4.8 | 0.005 | LOC107770581 | nuclear transcription factor Y subunit A-7-like |
| novel-nta-miR7-5p | -6.2 | 0.000 | LOC107770581 | nuclear transcription factor Y subunit A-7-like |
| novel-nta-miR87-5p | -1.1 | 0.000 | LOC107774584 | leucine-rich repeat receptor-like tyrosine-protein kinase PXC3 |
| novel-nta-miR89-3p | -3.7 | 0.018 | LOC107762814 | LOB domain-containing protein 21-like |
| novel-nta-miR96-5p | -7.4 | 0.007 | LOC107770581 | nuclear transcription factor Y subunit A-7-like |
| novel-nta-miR97-5p | -2.7 | 0.000 | LOC107761676 | probable LRR receptor-like serine/threonine-protein kinase |
| nta-miR156b | -24.5 | 0.000 | LOC107760247 | squamosa promoter-binding protein 1-like |
| nta-miR166a | -9.2 | 0.004 | LOC107760551 | homeobox-leucine zipper protein ATHB-15-like |
| nta-miR166c | -9.6 | 0.001 | LOC107760551 | homeobox-leucine zipper protein ATHB-15-like |
| nta-miR167d | -6.2 | 0.022 | LOC107766225 | protein DETOXIFICATION 49-like |
| nta-miR168c | -10.9 | 0.000 | LOC107817105 | protein RBL-like |
| nta-miR169a | -5.7 | 0.005 | LOC107770581 | nuclear transcription factor Y subunit A-7-like |
| nta-miR169h | -21.6 | 0.000 | LOC107770581 | nuclear transcription factor Y subunit A-7-like |
| nta-miR319b | -7.1 | 0.038 | LOC107770063 | transcription factor GAMYB-like |
| nta-miR399e | -8.2 | 0.046 | LOC107779704 | inorganic phosphate transporter 1-4-like |
| nta-miR482b-3p | -8.8 | 0.003 | LOC107760541 | putative late blight resistance protein homolog R1A-10 |
| nta-miR5303a | -8.0 | 0.000 | LOC107760705 | F-box/kelch-repeat protein At1g55270-like |
| nta-miR6149a | -9.4 | 0.001 | LOC107773429 | F-box/kelch-repeat protein At3g06240-like |

**Table S3. List of miRNAs up-regulated under drought treatment (D VS control)**

| **Gene ID** | **log_2_ (D / CK)** | **Q value** | **Target Gene ID** | **Annotation** |
| --- | --- | --- | --- | --- |
| novel-nta-miR27-3p | 1.37 | 0.014 | LOC107768414 | pentatricopeptide repeat-containing protein At1g22960, mitochondrial-like |
| novel-nta-miR305-5p | 1.17 | 0.036 | LOC107776266 | G-type lectin S-receptor-like serine/threonine-protein kinase At4g27290 |
| novel-nta-miR333-3p | 9.44 | 0.001 | LOC107769236 | ABC transporter G family member 32-like |
| nta-miR156c | 7.66 | 0.005 | LOC107760247 | squamosa promoter-binding protein 1-like |
| nta-miR156e | 25.35 | 0.000 | LOC107760247 | squamosa promoter-binding protein 1-like |
| nta-miR164a | 11.40 | 0.038 | LOC107762763 | NAC domain-containing protein 21/22-like |
| nta-miR166d | 11.01 | 0.000 | LOC107760551 | homeobox-leucine zipper protein ATHB-15-like |
| nta-miR394 | 6.12 | 0.018 | LOC107786252 | probable methyltransferase PMT22 |
| nta-miR395c | 8.74 | 0.016 | LOC107773366 | ATP sulfurylase 1 |
| nta-miR399g | 22.48 | 0.000 | LOC107779704 | inorganic phosphate transporter 1-4-like |
| nta-miR479b | 21.12 | 0.000 |  |  |
| nta-miR482c | 7.89 | 0.005 | LOC107775023 | TMV resistance protein N-like |
| nta-miR5303b | 7.74 | 0.000 | LOC107760705 | F-box/kelch-repeat protein At1g55270-like |
| nta-miR6019b | 10.42 | 0.010 | LOC107800887 | TMV resistance protein N-like |
| nta-miR6020a-5p | 10.95 | 0.001 | LOC107800887 | TMV resistance protein N-like |
| nta-miR6154a | 13.56 | 0.000 | LOC107800019 | protein REDUCED WALL ACETYLATION 2 |

| **Gene ID** | **log_2_ (DS / D)** | **Q value** | **Target Gene ID** | **Annotation** |
| --- | --- | --- | --- | --- |
| novel-nta-miR109-5p | -10.41 | 3E-06 | LOC107764512 | zinc finger CCCH domain-containing protein 19-like |
| novel-nta-miR11-3p | -8.39 | 7E-03 | LOC107759671 | scarecrow-like protein 15 |
|  |  |  | LOC107763536 | scarecrow-like protein 6 |
|  |  |  | LOC107763537 | scarecrow-like protein 6 |
| novel-nta-miR128-3p | -23.07 | 3E-07 | LOC107779704 | inorganic phosphate transporter 1-4-like |
| novel-nta-miR23-5p | -10.44 | 2E-02 | LOC107762327 | squamosa promoter-binding-like protein 4 |
| novel-nta-miR27-5p | -22.81 | 3E-07 | LOC107760285 | phosphatidylinositol 4-kinase gamma 4-like |
|  |  |  | LOC107810478 | putative late blight resistance protein homolog R1B-23 |
| novel-nta-miR294-5p | -7.98 | 6E-03 | LOC107760043 | importin-5-like |
| novel-nta-miR31-5p | -1.43 | 1E-05 | LOC107759323 | peroxisome biogenesis protein 6 |
|  |  |  | LOC107764805 | ABC transporter A family member 11-like |
| novel-nta-miR35-5p | -8.24 | 1E-02 | LOC107817105 | protein RBL-like |
| novel-nta-miR94-3p | -1.00 | 5E-03 | LOC107761123 | unknown |
| nta-miR156a | -25.04 | 2E-08 | LOC107770163 | serine/threonine protein phosphatase 2A 55 kDa regulatory subunit B beta isoform-like |
| nta-miR156c | -5.74 | 2E-02 | LOC107770163 | serine/threonine protein phosphatase 2A 55 kDa regulatory subunit B beta isoform-like |
| nta-miR156h | -11.15 | 2E-02 | LOC107762327 | squamosa promoter-binding-like protein 4 |
|  |  |  | LOC107770163 | serine/threonine protein phosphatase 2A 55 kDa regulatory subunit B beta isoform-like |
| nta-miR160c | -10.65 | 3E-03 | LOC107760064 | auxin response factor 18-like |
| nta-miR166h | -12.02 | 2E-08 | LOC107760551 | homeobox-leucine zipper protein ATHB-15-like |
| nta-miR398 | -8.97 | 3E-06 | LOC107769236 | ABC transporter G family member 32-like |
|  |  |  | LOC107829449 | blue copper protein-like |
| nta-miR399f | -24.75 | 2E-08 | LOC107779704 | inorganic phosphate transporter 1-4-like |
| nta-miR482c | -9.78 | 2E-04 | LOC107775023 | TMV resistance protein N-like |
| nta-miR6019b | -11.35 | 4E-02 | LOC107778652 | extensin-1-like |
|  |  |  | LOC107791820 | TMV resistance protein N-like |
| nta-miR6146a | -8.52 | 3E-03 | LOC107773212 | calmodulin-lysine N-methyltransferase-like |
|  |  |  | LOC107814327 | E3 ubiquitin-protein ligase MIEL1-like |
| nta-miR6154a | -13.54 | 3E-04 | LOC107800019 | protein REDUCED WALL ACETYLATION 2 |

**Table S4.** List of miRNAs down-regulated under drought+Se treatment (DS VS D)

**Table S5.** List of miRNAs upregulated under drought+Se treatment (DS VS D)

| **Gene ID** | **log2**  **(DS / D)** | **Q value** | **Target Gene ID** | **Annotation** |
| --- | --- | --- | --- | --- |
| novel-nta-miR109-3p | 1.29 | 7.14E-05 | LOC107827404 | E3 ubiquitin-protein ligase ATL41-like |
| novel-nta-miR110-3p | 2.01 | 2.88E-12 | LOC107762318 | protein transport protein Sec24-like At4g32640 |
| novel-nta-miR133-5p | 2.16 | 1.18E-02 | unkown | unkown |
| novel-nta-miR156-5p | 1.57 | 3.46E-03 | LOC107771768 | transcription factor bHLH30-like |
|  |  |  | LOC107796775 | probable metal-nicotianamine transporter YSL7 |
| novel-nta-miR19-3p | 1.83 | 3.12E-02 | LOC107768246 | unkown |
| novel-nta-miR209-5p | 1.91 | 1.34E-03 | LOC107808871 | serine/threonine-protein kinase RUNKEL |
| novel-nta-miR218-3p | 1.39 | 7.48E-03 | LOC107791237 | RNA polymerase II C-terminal domain phosphatase-like 4 |
| novel-nta-miR230-5p | 1.57 | 1.18E-02 | LOC107794394 | 60S ribosomal protein L6, mitochondrial-like |
| novel-nta-miR248-5p | 5.31 | 7.38E-03 | LOC107766225 | protein DETOXIFICATION 49-like |
| novel-nta-miR287-3p | 1.45 | 7.14E-05 | LOC107794633 | protein SRG1-like |
|  |  |  | LOC107804789 | CBL-interacting serine/threonine-protein kinase 5-like |
| novel-nta-miR345-3p | 8.86 | 1.02E-02 | LOC107764608 | SPX domain-containing membrane protein At4g22990-like |
| novel-nta-miR37-3p | 1.29 | 7.27E-04 | LOC107767188 | bifunctional riboflavin kinase/FMN phosphatase-like |
| novel-nta-miR378-3p | 2.12 | 4.09E-07 | LOC107790606 | cation/calcium exchanger 4-like |
| novel-nta-miR412-5p | 1.84 | 7.45E-10 | LOC107794394 | 60S ribosomal protein L6, mitochondrial-like |
| novel-nta-miR419-3p | 1.58 | 4.70E-02 | LOC107768388 | ultraviolet-B receptor UVR8-like |
| novel-nta-miR97-5p | 1.49 | 1.69E-02 | LOC107761676 | probable LRR receptor-like serine/threonine-protein kinase At4g20940 |
| nta-miR156b | 22.82 | 3.20E-07 | LOC107762327 | squamosa promoter-binding-like protein 4 |
|  |  |  | LOC107770163 | serine/threonine protein phosphatase 2A 55 kDa regulatory subunit B beta isoform-like |
| nta-miR156d | 10.55 | 7.48E-03 | LOC107762327 | squamosa promoter-binding-like protein 4 |
|  |  |  | LOC107770163 | serine/threonine protein phosphatase 2A 55 kDa regulatory subunit B beta isoform-like |
| nta-miR156i | 11.24 | 6.92E-03 | LOC107762327 | squamosa promoter-binding-like protein 4 |
|  |  |  | LOC107770163 | serine/threonine protein phosphatase 2A 55 kDa regulatory subunit B beta isoform-like |
| nta-miR162b | 6.19 | 4.70E-02 | LOC107786433 | auxin-responsive protein SAUR71-like |
| nta-miR166a | 9.44 | 3.32E-03 | LOC107760551 | homeobox-leucine zipper protein ATHB-15-like |
| nta-miR166b | 8.46 | 1.23E-02 | LOC107760551 | homeobox-leucine zipper protein ATHB-15-like |
| nta-miR390a | 10.55 | 1.23E-02 | LOC107774584 | leucine-rich repeat receptor-like tyrosine-protein kinase PXC3 |
| nta-miR395b | 13.32 | 9.38E-03 | LOC107773366 | ATP sulfurylase 1 |
| nta-miR396b | 8.92 | 1.95E-03 | LOC107760378 | putative B3 domain-containing protein At5g35780 |
|  |  |  | LOC107764512 | zinc finger CCCH domain-containing protein 19-like |
| nta-miR399c | 8.82 | 1.16E-02 | LOC107779704 | inorganic phosphate transporter 1-4-like |
| nta-miR482b-3p | 9.65 | 4.71E-05 | LOC107760541 | putative late blight resistance protein homolog R1A-10 |
| nta-miR6154b | 11.99 | 6.61E-03 | LOC107800019 | protein REDUCED WALL ACETYLATION 2 |

**Table S6.** Links between the drought tolerance-related metabolites and miRNAs and their target genes

| miRNA | Target gene ID | Annotation | Metabolite | Correlation | P value |
| --- | --- | --- | --- | --- | --- |
| novel-nta-miR156-5p | LOC107796775 | metal-nicotianamine transporter YSL7 | Phenyl hydrogen sulfate | + | <0.001 |
|  |  |  | Lactose | - | <0.001 |
| novel-nta-miR97-5p | LOC107761676 | LRR receptor-like serine/threonine-protein kinase | Butabarbital | - | <0.001 |
|  |  |  | Catechin | - | <0.001 |
|  |  |  | Oglufanide | - | <0.001 |

+ and – represent positive and negative correlation.
